# Polymorphisms on SSC15q21-q26 Containing QTL for reproduction in Swine and its association with litter size

**DOI:** 10.1590/S1415-47572009000100010

**Published:** 2009-03-01

**Authors:** Hongli Du, Jing Chen, Jianxun Cui, Xiaoning Wang, Xiquan Zhang

**Affiliations:** 1 School of Bioscience and Bioengineering, South China University of Technology, GuangzhouChina; 2 College of Animal Science, South China Agricultural University, GuangzhouChina

**Keywords:** porcine, reproductive traits, NRP2, candidate gene

## Abstract

Several quantitative trait loci (QTL) for important reproductive traits (ovulation rate) have been identified on the porcine chromosome 15 (SSC15). To assist in the selection of positional candidate swine genes for these QTL on SSC15, twenty-one genes had already been assigned to SSC15 in a previous study in our lab, by using the radiation hybrid panel IMpRH. Further polymorphism studies were carried out on these positional candidate genes with four breeds of pigs (Duroc, Erhualian, Dahuabai and Landrace) harboring significant differences in reproduction traits. A total of nineteen polymorphisms were found in 21 genes. Among these, seven in six genes were used for association studies, whereby NRP2 polymorphism was found to be significantly (p < 0.05) associated with litter-size traits. NRP2 might be a candidate gene for pig-litter size based on its chromosome location (Du *et al.*, 2006), significant association with litter-size traits and relationships with Sema and the VEGF super families.

## Introduction

More than 1800 quantitative trait loci (QTL) had already been mapped on the entire pig genome until June 3^rd^, 2008, based on statistics available in the Pig QTL Database. The next step would be to identify candidate genes for these traits by developing a detailed comparative map and a SNP map that would both constitute an effective method for assisting in the selection of the underlying genes responsible for the mapped QTL. For example, a QTL affecting meat quality in chromosome 2p has already been well described (Andersson-Eklund *et al.* 1998; Knott *et al.* 1998; de Koning *et al.* 1999), these methods being essential in the identification of regulatory mutation in the causal gene IGF-2 (Jeon *et al.* 1999; Nezer *et al.* 1999; de Koning *et al.* 2000; Nezer *et al.* 2003; Van Laere *et al.* 2003).

Single nucleotide polymorphisms (SNP) are far more abundant, with the occurrence of about one SNP per 1000 bp in human beings (Wang *et al.* 1998) and one SNP per 200 bp in chickens (International Chicken Polymorphism Map Consortium, 2004), and are more amenable to assessment on using high-throughput genotyping technologies. Furthermore, SNP are extremely stable, occurring at a rate of only one mutation in 2 x 10^8^ nucleotides in humans (Sachidanandam *et al.* 2001). A straightforward strategy for the identification of SNP is locus-specific amplification (LSA) and comparative re-sequencing from multiple individuals (Rieder *et al.* 1998). In addition, knowledge of local linkage disequilibrium (LD) and common haplotype patterns in disease association and positional cloning studies is becoming increasingly widespread since it has become clear (Rioux *et al.* 2001; Van Eerdewegh *et al.* 2002; Geesaman *et al.* 2003; Stoll *et al.* 2004) that intelligent use of this information has the potential of making them much more comprehensive and efficient. LD plays a fundamental role in the fine mapping of QTL and in proposed genome-wide association studies (Nezer *et al.* 2003).

QTLs for the ovulation rate (OR) were detected in the porcine chromosome 15q21-q26 (Rathje *et al.* 1997; Rohrer *et al.* 1999). The identified region spans over 60 cM, which corresponds to chromosomal fragments containing hundreds of genes. In a previous study in our lab, twenty-one genes were assigned to SSC15 by using the radiation hybrid panel IMpRH (Du *et al.*, 2006). The goal of the present study was to screen SNP in these genes and investigate their association with litter size for identifying candidate genes in this QTL region. Seven polymorphisms were chosen for investigating their effects on litter size by using animals (unrelated individuals) which had already been recorded for litter-size traits.

## Materials and Methods

### Pig populations

Ear samples from 237 domestic pigs from the four breeds, Duroc, Erhualian, Dahuabai and Landrace, numbering 38, 58, 61 and 80 individuals, respectively, were randomly collected from the Guangdong Wenshi Foodstuff Co. Ltd. (Guangdong, China), Guangdong Changjiang Foodstuff Company Ltd. (Guangdong, China) and Guangdong Banling Pig Farm (Guangdong, China). One hundred and fifty eight individuals were recorded as originating 4 or more than 4 litters. A fertility index was calculated individually by litter size, prenatal survival and litter-weight for all the litters of the 158 animals and assigned to three parameters: litter size (LS), prenatal survival (PS), litter-weight (LW).

### Genomic DNA pool preparation

Porcine genomic DNA was extracted from ear samples using the Nucleon kit (TaKaRa, Japan). DNA concentrations were determined by O.D. measurements, using a Biophotometer (Eppendorf, Germany). All samples were diluted to a concentration of 20 ng/μL. The breed DNA pool was constructed by mixing 30 samples from each breed at equal volume, whereby four breed DNA pools (Duroc, Erhualian, Dahuabai and Landrace) were obtained.

### PCR and sequencing

The 21 primer pairs (Table 1) were also used to amplify genomic DNA fragments with the four breed DNA pools described above. Direct sequencing of these PCR products was performed using an ABI 377 automatic sequencer (Applied Biosystems, Foster City, Calif., USA) following standard protocols. The sequence for each single gene was subjected to a BLAST search to confirm both its origin and ortholog status.

PCR reactions were performed in a final volume of 25 μL containing 50 ng of porcine genomic DNA, 0.4 μM of each primer, 200 μM of each dNTPs, 1.5 mM MgCl_2_, 1 x Taq buffer, and 0.7 U of *Taq* polymerase (TaKaRa, Tokyo, Japan.). PCR conditions were as follows: 94 °C for 5 min and 35 cycles of 30 s at 94 °C, 30 s at annealing temperature (Table 1) and 30 s at 72 °C, followed by a final 5 min extension at 72 °C. PCR products were then examined by electrophoresis on a 2% agarose gel stained with ethidium bromide and then photographed.

### Polymorphism identification and selection

Sequencing gel images obtained from previous experiments were analyzed using Sequencing Analysis software (Applied Biosystems, Foster City, Calif., USA) for lane tracking and trace file extraction. Subsequently, all trace files were analyzed using the Pregap4 program of the Staden software package (Bonfield and Staden 1996; http://www.mrc-lmb.cam.ac.uk/pubseq). All nucleotide positions at which disagreement occurred were tagged as putative polymorphism positions. Sequences were submitted to the GenBank. Polymorphisms identified in the sequences are represented by the IUB ambiguity codes reflecting alleles found in the DNA pools.

In addition, the preliminary allele distribution of each polymorphism in each breed could be estimated based on peak heights at the polymorphic site in trace files (Cui *et al.* 2005). The effect of these markers on reproduction traits could be deduced to some extent, due to the diverse allelic distribution in different breeds. According to this principle, the seven polymorphisms identified in six genes (DPP4, SH3BP4, ORC2L, NRP2, COL4A4 and TRTP12) were selected and genotyped by the PCR-RFLP method, their effects on litter size being investigated by using unrelated individuals wherein the trait of litter size had already been recorded.

### Genotyping, haplotype construction and allelic frequency

A total of 237 individuals from the four breeds Duroc, Erhualian, Dahuabai and Landrace, numbering 38, 58, 61 and 80 individuals, respectively, were genotyped by the PCR-RFLP method at the seven polymorphism sites, selected as above. Fragments of DPP4, SH3BP4, ORC2L, NRP2, and TRTP12 were digested by the *Cfr*I*, Xap*I*, BseM*I, *Eco105*I and *Bsh1236*I endonucleases, respectively, and the COL4A4 fragment by the *TasI* and *NdeI* endonucleases, to be then electrophoresed on 3% agarose gel for genotyping. The allelic frequencies of the seven polymorphisms were calculated in all the populations.

Haplotypes were constructed with two SNP for COL4A4 in all experimental animals, through applying the PHASE programme (Stephens *et al.* 2001), with the reconstruction of haplotypes from population data as the main function. The minimum haplotype frequency was set at 2%.

### Linkage disequilibrium analyses

In this study, LD analyses were performed using HAPLOVIEW software (Barrett *et al.* 2005) in a population of 237 individuals, and LD measured with a parameter of r^2^ (Ardlie *et al.* 2002).

### Marker-trait association analysis

The association of single polymorphism or haplotype with litter size was analyzed by means of the GLM procedure of SAS 8.0 (Statistical Analysis Systems Institute Inc., Cary, NC, USA). The model included parity, genotype (or haplotype), farm and breed as fixed effects for all the 158 animals. In addition, an association study was also carried out for each breed. Values were considered significant at p < 0.05 and presented as least square means (LSM) ± standard error means (SEM).

## Results

### Gene sequencing

Direct sequencing of the 21 gene PCR products in both directions yielded reliable and readable sequences for pooled DNA samples from Duroc, Erhualian, Dahuabai and Landrace pigs.

### Polymorphism identification

Direct sequencing of the 21 gene PCR products in both directions yielded 168 readings, all of which being assembled in 21 distinct contigs for the four pooled DNA samples. Contigs varied in length between 220 and 748 bp, with an average of 429 bp, and a total length of 8 943 bp. The assembled sequences were submitted to GenBank, and accepted with the uninterrupted accession numbers from AY805665 to AY805748 (Table 1). At least one polymorphism was identified in 11 genes (COL4A4, DPP4, EPHA4, HES6, HSPE1, KCNJ3, NRP2, ORC2L, SH3BP4, SP3 and TRIP12) among the 21 genes analyzed. We identified 19 polymorphic positions in the total contig length of 8 943 bp, corresponding to an overall average of one polymorphism per 470 bp. Most of these, 14 out of the19, are located in the SSC15 q23-q25 region. Detailed information on each polymorphism is available in the submitted sequences.

### Genotypes, haplotypes and their frequencies

Three haplotypes were found at each site of six genes (DPP4, SH3BP4, ORC2L, NRP2, COL4A4 and TRTP12). Large breed differences were observed in the allelic frequencies of 6 SNPs and 4-bp indel (Table 2). The most significant differences were found between the Duroc and Erhualian breeds at the T-SH3BP4-G, T-NRP2-C and C-COL4A4-2-T sites.

Haplotypes constructed based on two SNPs of the COL4A4 gene and their frequencies in four populations, are shown in Table 3. Four haplotypes were identified in 237 individuals, in which the three most abundant, designated H1 (C T), H2 (C A) and H3 (T A), accounted for 98% of frequency. Haplotype H4 (T T) seems to correspond to a recombination between H1 and H3 and is present only in Duroc pigs. Their frequencies are significantly different among the four breeds.

### LD and marker-trait association

The r^2^ value was estimated for each of two polymorphic sites, the highest r^2^ value (0.59) being obtained between the ORC2L and NRP2 polymorphic sites and the two loci forming a haplotype block.

The effect of single polymorphism or haplotype on reproduction traits in pigs was estimated simultaneously for the parameters of LS, PS and LW. We found that NRP2 genotypes had a significant effect on litter-size traits (type III F = 5.18, p = 0.0059 for LS; type III F = 5.70, p = 0.0035 for PS; type III F = 9.17, p = 0.0001 for LW), whereas all the other genotypes at any other polymorphism site had no effect whatsoever on reproduction traits. The significant effect of NRP2 genotypes can also be inferred by association analysis which was carried out for Dahubai and Landrace individuals, with the SAS GLM procedure including parity and genotype (or haplotype) as fixed effects (Table 4).

## Discussion

In our previous study (Du *et al.*, 2006), all the genes except ARHE, SH3BP4 and HES6 had been mapped within the QTL regions at 53-101 cM, and 79.3-102.5 cM characterized for the ovulation rate (Pig QTL Database, QTL on Pig Chromosome 15). Therein, KLF7, NRP2 and ORC2L were located very close to the QTL center position (79 cM) (Rohrer *et al.* 1999), as the adjacent microsatellites SW1316, SWR1002 and SW2083 were mapped at 73.1 cM, 76 cM and 81.1 cM, respectively, on the MARC genetic map, all available at http://www.animalgenome.org/maps/marc.html. Similarly, EPHA4, COL4A4 and TRIP12 were located near to the QTL center position (88.5 cM) (Rathje *et al.* 1997), deduced from the position of SW936, SW906 and SW2608 mapped at 88.5 cM, 89.3 cM and 95 cM respectively on the MARC genetic map. Therefore, these six genes are the most possible positional candidate genes among the 21 genes analysed.

Erhualian is one of the Taihu breeds with the highest fertility, producing around 16 piglets all told and 14 live piglets on an average per litter. Dahuabai is a native Chinese breed which produces about 14 piglets overall and 13 live piglets on an average per litter. Landrace and Duroc are breeds with a rapid growth rate, but producing far fewer piglets per litter than native Chinese breeds do. The four breeds were investigated to screen for potential polymorphisms related to reproductive traits in SSC15 based on DNA pooling and sequencing due to their very different litter sizes. Seven polymorphisms in the six genes DPP4, SH3BP4, ORC2L, NRP2, COL4A4 and TRTP12 were selected and genotyped for 237 individuals for further LD and marker-trait association studies, based on approximate allele distribution in the four breeds. Allelic frequency studies revealed that the frequencies of T-SH3BP4, T-NRP2 and C-COL4A4-2 varied significantly among the four breeds, the most significant differences being found especially between the Duroc and Erhualian breeds at the three sites. However, only the significant association of T-NRP2-C with litter size traits (type III F = 5.18, p = 0.0059 for LS; type III F = 5.70, p = 0.0035 for PS; type III F = 9.17, p = 0.0001 for LW) was observed when further association studies were carried out on each polymorphism (Table 4). The over dominance expressions of T-NRP2-C for the PS trait are also to be found in Table 4. The significant difference between TC and CC or TT for traits can also be observed in the Landrace and Dahuabai breeds, which can validate the results for all the animals to some extent.

The association of NRP2 with litter-size traits is consistent with the QTL which was characterized for the ovulation rate (Rohrer *et al.* 1999), since the latter is a major factor for litter size. Neuropilin (NRP, previously referred to as A5) is a type I transmembrane protein which is widely distributed in vertebrate species such as Xenopus, chickens and mice (Fujisawa *et al.* 1995). The extracellular part of the neuropilin protein is composed of three unique domains, each of which is thought to be involved in molecular and/or cellular interactions (Kitsukawa *et al.* 1995). NRP2 is a member of the related neuropilin family.. It is a receptor for the secreted semaphorins Sema IV and Sema E, and acts selectively to mediate repulsive guidance events in discrete populations of neurons (Chen *et al.* 1997). It has also been found to be a receptor for the vascular endothelial growth factor (VEGF) forms VEGF-145 and VEGF-165 and for additional VEGF family-members, such as the placenta growth factor (PlGF-2) (Gluzman-Poltorak *et al.* 2000; Neufeld *et al.* 2002). VEGF induces endothelial cell proliferation, promotes cell migration and inhibits apoptosis. A recent study has revealed that NRP2 is probably related to lymphangiogenic growth (Lohela *et al.* 2003) and a deficiency in NRP2 suppresses VEGF-induced retinal neovascularization (Shen *et al.* 2004). In conclusion, NRP2 can be considered as a candidate gene for litter-size traits based on its chromosome location, significant association with litter-size traits and relationships with the Sema and VEGF super families.

## Figures and Tables

**Table 1:** Information for PCR amplification of the genes genotyped on the IMpRH panel.

Gene name	Primers (forward/ reverse)	Tm (°C)	Porcine sequence Acc. n.	Length (bp)
ACVR1	TGGTGTCACATGGGTAATGG/GGCTGCATACTCCAACTGGT	60	AY805729	395
ARHE	GACCTTTATATTGTTGGCGC/ACTGCAACGAAAGTTCTGCT	56.5	AY805745	341
CED-6	CCCAACATCCACTGTAGATC/TGCCATCTGTCTCATGAGTG	58	AY805705	324
CMYA3	GCAATGCCCTCTTCAGATAC/CAGCAGTACAGAATGTCACC	58.5	AY805685	690
COL4A4	CATACCTTGCCACTGGGTTG/ATCTCTTTGCCTTCCCCTGA	60	AY805733	305/303
DPP4	ATGGCAGGATTTGGGCAGTG/CAGGGCAAGCTGATGTGTTC	61	AY805665	420
EPHA4	GTGGTATCGGTGGGTGATTG/TTCTGCCGTGTATCTGTTGC	61	AY805669	553/552
FLJ11457	AAGGCCCGTGCATGTTTAAG/CCCGGAAGTTTACTCAAGTC	58.5	AY805681	438
GAD1	AGTCACTTTACCTTCAGCGG/GCAGATGCTATGCAAGTGTG	58.5	AY805689	664
HAT1	TGTGGAAGATTATCGACGTG/CAGTGGTATTTCAGAAGCTC	58.5	AY805693	268
HES6	AGAGGATGGCTTGCCGTGAA/ATGCAAGACATACAGGGAGC	60	AY805725	383
HSPE1	GGCAGTACTGCAGGTCTTTC/CTGATCCAACAGCTACTACG	58	AY805709	748/747
KCNJ3	TGGTCCCTATTTCAACTGCG/TGTTAGGCCAGAGATCTAGG	61	AY805673	394
KLF7	TTCTCTCGACAGCTACACGG/TTCTTGTTTTCGGGGCATGC	60	AY805737	297
NFE2L2	AGCCTTACTCTCCAAGTGAG/ACGTATACAGAAACTAGCCC	58	AY805713	349
NR4A2	AGCTCCCAGAACTTCGTACG/GTCCCCTTACAATGTGGGTG	58.5	AY805701	554
NRP2	CTCTGGCTGACAAGGAGAAG/CGTCAGATGTGATGTGGGTG	60	AY805721	275
ORC2L	GGAAGTTTGAAGCTCTCCTA/CTGACATACGGAATAGCAGA	56.5	AY805717	476/472
SH3BP4	CATGGACCAGGACGACTATG/GCATCTGCCCCTATGAAGTG	58.5	AY805697	418/417
SP3	AAACCCATATGTCTGCCAGG/AGGTGAACTGCTGCAGTTAC	62	AY805677	431
TRIP12	GAAGCTGTTGATGGCGGCAA/GGGGCCATGAATGAATATGA	60	AY805741	220

**Table 2:** Allele frequencies of the 7 polymorphic sites in 4 pig populations and Pearson's χ^2^ value.

Breeds (n. pig)^a^	DPP4-1			SH3BP4			ORC2L			NRP2			COL4A4-2			COL4A4-3			TRTP12	
	C	T		T	G		-	GTAC		T	C		C	T		T	A		G	A
Duroc (38)	0	1.00		0	1		0	1.00		0	1.00		0.46	0.54		0.51	0.49		0.46	0.54
Erhualian (58)	0.29	0.71		1.00	0		0.35	0.65		0.70	0.30		1.00	0		0.21	0.79		0.59	0.41
Dahuabai (61)	0.52	0.48		0.96	0.04		0.70	0.30		0.66	0.34		0.93	0.07		0.53	0.47		0.28	0.72
Landrace (80)	0.04	0.96		0.14	0.86		0	1.00		0.04	0.96		0.76	0.24		0.31	0.69		0.13	0.87
χ^2^_6_^b^	105.77			234.41			186.33			203.80			103.35			57.80			66.04	
Acc. n.^c^	AY805665			AY805697			AY805717			AY805721			AY805733			AY805733			AY805741	
Variation position	Pos 296			Pos 242			Pos 187			Pos 62			Pos 147			Pos 188			Pos 69	

^a^The number in brackets is the individual number investigated in each breed. ^b^χ^2^_6_ > 16.81, p < 0.01. ^c^Porcine sequence accession numbers.

**Table 3:** Base constitutions of COL4A4 haplotypes and their frequencies.

haplotype	Sites			Frequencies			
	COL4A4-2	COL4A4-3		Duroc(38)	Erhualian(58)	Dahuabai (61)	Landrace (80)
h1	C	T		0.43	0.79	0.53	0.31
h2	C	A		0.03	0.21	0.40	0.45
h3	T	A		0.46	0	0.07	0.24
h4	T	T		0.08	0	0	0

^a^The number in brackets is the individual number investigated in each breed.

**Table 4:** Least square means (LSM ± SEM) of Litter size (LS), Pre-natal survival (PS) and Litter weight (LW), according to *NRP2* polymorphism.

Trait^b^	All 158 animals									Landrace						Dahuabai							
	Genotype^a^				Type III p value					Genotype			Type III p value			Genotype				Type III p value			
	CC(102)	TC (36)	TT(20)		Total	CC/TC	CC/TT	TC/TT		CC(72)	TC (6)		Total	CC/TC		CC(1)	TC (30)	TT(20)		Total	CC/TC	CC/TT	TC/TT
LS	11.18 ± 0.35	12.20 ± 0.43	11.27 ± 0.53		0.0059*	0.0557	0.8850	0.0094*		10.79 ± 0.52	11.88 ± 0.75		0.0585	0.0585		13.12 ± 2.77	13.09 ± 0.32	12.18 ± 0.37		0.0420*	0.9923	0.7363	0.0120*
PS	10.38 ± 0.35	11.52 ± 0.43	10.59 ± 0.53		0.0035*	0.0320*	0.7443	0.0088*		9.71 ± 0.49	10.96 ± 0.70		0.0202*	0.0202*		13.14 ± 3.1	12.96 ± 0.36	12.05 ± 0.42		0.0816	0.9537	0.7279	0.0256*
LW (kg)	11.13 ± 0.37	13.44 ± 0.46	13.00 ± 0.56		0.0001*	< .0001*	0.0056*	0.2344		13.35 ± 0.67	15.84 ± 0.96		0.0008*	0.0008*		7.59 ± 1.91	7.22 ± 0.22	6.80 ± 0.26		0.2312	0.8471	0.6808	0.0911

^a^The number in brackets is the observed number of genotype; therein, one CC, 30 TC and 20 TT from Dahuabai, 29 CC from Duroc, 72 CC and 6 TC from Landrace. *p ≤ 0.05.
